# Burning health inequity: blood pressure effects of household cooking with solid fuels among Chinese women

**DOI:** 10.3389/fpubh.2025.1683629

**Published:** 2025-12-12

**Authors:** Qian Liu, Rui Wang

**Affiliations:** School of Public Administration and Policy, Shanghai University of Finance and Economics, Shanghai, China

**Keywords:** health inequity, household air quality, solid fuel, blood pressure, women’s health

## Abstract

**Introduction:**

Although modern energy is widely available in China, many households still cook with solid fuels, generating substantial household air pollution and posing a significant public health risk.

**Methods:**

Using nationally representative CNHS data from 30,844 women, this study examined the association between solid-fuel cooking and blood pressure, with effects estimated using individual fixed-effects models.

**Results:**

On average, women who cook with solid fuels have a 0.548 mmHg higher SBP than those who use clean fuels. Subgroup analyses show stronger adverse associations among urban residents, employed women, and older adults, suggesting an elevated risk of hypertension in these groups.

**Discussion:**

Heterogeneity results further suggest that poor housing conditions may exacerbate these effects. Although the average effect size is modest, the overall evidence indicates adverse health impacts from solid-fuel use and highlights the need for targeted intervention strategies.

## 1 Introduction

Air quality is closely linked to human health, and its improvement has become a global policy priority. China, for example, launched nationwide efforts to reduce coal consumption in 2013. Although industrial emission controls are widely viewed as pivotal for reducing pollution-related diseases, evidence on the individual-level health impacts of indoor air pollution remains incomplete. Numerous epidemiological studies have linked household air pollution to a wide range of serious health outcomes, yet important gaps persist in quantifying these effects and identifying vulnerable subpopulations (1–5). According to the Global Burden of Disease Study (1990–2016), indoor air pollution from solid-fuel use ranks among the top behavioral risk factors and is among the top 10 risk factors overall (6). Accumulating evidence indicates that the health impacts of indoor solid-fuel use are substantial, warranting urgent attention and targeted, evidence-based interventions.

This study examines the association between solid-fuel cooking and blood pressure among Chinese women. The primary exposure is household solid-fuel use—coal or biomass (e.g., wood, crop residues, and dung) for cooking or heating—with liquefied petroleum gas (LPG) treated as a clean-fuel comparator (7–9). Blood pressure serves as the study’s dependent variable and primary health indicator for two reasons. First, although hypertension is widely recognized as one of the top three global health risks (6), the impact of household solid-fuel use on blood pressure remains insufficiently characterized. Second, hypertension is often asymptomatic yet can cause severe cardiac and renal damage; cardiovascular disease attributable to elevated blood pressure accounts for approximately 10.8 million deaths annually (10, 11).

As of 2021, the World Health Organization estimated that 2.3 billion people still relied on polluting fuels and technologies for household energy (12). Combustion of solid fuels releases substantial quantities of pollutants, including particulate matter, carbon monoxide (CO), and sulfur dioxide (SO₂) (13). Among these, CO and SO₂ are colorless, toxic gases. Carbon monoxide (CO) impairs the blood’s oxygen-carrying capacity by binding to hemoglobin to form carboxyhemoglobin. Prolonged exposure to elevated CO levels can raise blood pressure and may increase the risk of cardiovascular disease (14–16). Particulate matter (PM) comprises fine solid and liquid particles suspended in the air that can cause substantial adverse health effects when inhaled. Two primary pathways have been proposed through which PM may influence blood pressure (17). First, inhaled particulate matter (PM) can disturb autonomic nervous system balance, leading to elevated blood pressure. Second, PM deposited in the lungs may translocate into the circulation, where it promotes systemic arterial vasoconstriction, thereby increasing blood pressure (18). Clinical and experimental studies consistently link higher ambient particulate matter (PM) concentrations with elevated blood pressure (17, 19).

Prior studies indicate that household solid-fuel use is associated with elevated blood pressure (20–24). Table A1 summarizes recent studies. The first panel focuses on the effects of specific pollutants on blood pressure. For example, Baumgartner et al. (25) reported that a one-unit increase in the natural log of black carbon concentration (μg/m^3^) was associated with, on average, a 4.3 mmHg higher systolic blood pressure (SBP) and a modest rise in diastolic blood pressure (DBP) among women. The authors further noted that women aged ≥50 years and those at elevated risk of hypertension (e.g., individuals with obesity or pre-existing high blood pressure) were particularly susceptible to the adverse effects of household air pollution (26–29).

The latter half of Table A1 synthesizes studies examining associations between household fuel or stove type and blood pressure. For instance, McCracken et al. (30) reported that adoption of improved cookstoves in Guatemala was associated with reductions in systolic and diastolic blood pressure (SBP, DBP) among women. Likewise, Alexander et al. (28) observed that switching from traditional high-emission fuels (e.g., wood, kerosene) to cleaner alternatives (e.g., ethanol) was associated with lower diastolic blood pressure among women in mid-to-late pregnancy.

The study found that the impact of using solid fuels on blood pressure is minimal (3, 16, 28, 29). Clark et al. (3) suggested that changing fuel type did not cause significant changes in blood pressure. Fewer studies have examined the association between fuel type and blood pressure in the Chinese context. Baumgartner et al. (31, 32) argued that the use of solid fuels makes systolic blood pressure rise in women. Similarly, Lee et al. (24), Yan et al. (23), and Shi et al. (20) observed similar trends across different datasets.

This study differs from prior research (23, 29, 33) in several respects. First, most previous analyses have relied on partial samples or single waves of the China Health and Nutrition Survey (CHNS). In contrast, this study used seven CHNS waves spanning multiple years to assess the overall impact of fuel switching on women’s health in China. For instance, Yan et al. (23) analyzed the 2009 wave. Likewise, although Nie et al. (29) utilized a larger CHNS sample, their analysis was restricted to rural women and focused on a limited set of fuels [e.g., wood/straw, coal, and liquefied petroleum gas (LPG)]. Prior studies have often restricted their samples to a single survey wave or to specific subgroups of fuel users and therefore do not provide a comprehensive assessment of the health effects of household solid-fuel use among Chinese women. Second, this study employed individual fixed-effects models to mitigate potential bias from time-invariant unobserved factors; by incorporating individual fixed effects, concerns about omitted-variable bias arising from stable individual characteristics were minimized. Third, the analysis controlled for multi-fuel use within households. In many developing countries, households commonly use multiple fuels simultaneously; for example, some primarily use clean fuels for cooking but still rely on solid fuels as a secondary energy source for heating or other purposes. Failure to account for secondary (multi-fuel) use may bias estimates in prior studies. This study examined how employment status, age, and place of residence influenced blood pressure among solid-fuel users. Prior research has typically considered these factors in isolation and has not fully explored their shared mechanisms. Using detailed heterogeneity analyses, we identified and explained these differences and informed targeted policy recommendations.

## 2 Data and methods

Data for this study were drawn from the China Health and Nutrition Survey (CHNS), a longitudinal household survey administered by the Carolina Population Center at the University of North Carolina at Chapel Hill in collaboration with the Chinese Center for Disease Control and Prevention. The analysis used seven waves conducted between 1997 and 2015 (1997, 2000, 2004, 2006, 2009, 2011, 2015); earlier waves (1989, 1991, 1993) did not collect information on secondary fuel use and were therefore excluded. The CHNS sample included more than 30,000 individuals in approximately 7,200 households across 15 provinces—Guangxi, Guizhou, Heilongjiang, Henan, Hubei, Hunan, Jiangsu, and Liaoning, and others. After excluding observations with implausible height or weight values (34), individuals under the age of 16, students, and non-residents (defined as individuals not living in households), the final analytic sample comprised 30,844 female observations. The exclusion of these groups was based on the fact that they do not engage in household cooking. The CHNS data are publicly available and de-identified; therefore, this secondary analysis did not require additional ethical approval or informed consent.

### 2.1 Blood pressure

Diastolic (DBP) and systolic (SBP) blood pressure were measured three times per participant in the China Health and Nutrition Survey (CHNS). For this analysis, the mean of the second and third readings was used. The first reading was excluded because initial measurements are typically higher when multiple readings are taken (35).

In addition to continuous blood pressure measures, the analysis constructed three binary indicators: systolic blood pressure (SBP) ≥ 110 mmHg, SBP ≥ 140 mmHg, and hypertension status. Hypertension was defined according to the Chinese Center for Disease Control and Prevention (China CDC) as diastolic blood pressure (DBP) ≥ 90 mmHg or systolic blood pressure (SBP) ≥ 140 mmHg; individuals with a prior diagnosis of hypertension or using antihypertensive medication were also classified as hypertensive. Robustness checks indicated that reclassifying hypertension according to the American Heart Association (AHA) criteria did not materially alter the findings. The SBP ≥ 110 mmHg indicator was included because 110 mmHg is often cited as a threshold associated with elevated cardiovascular and renal risk (36).

### 2.2 Exposure to indoor air pollution

Respondents reported their primary cooking fuel. Those who primarily used coal, charcoal, wood, or other biomass (e.g., sticks, straw) were classified as solid-fuel users; those whose primary fuel was kerosene, liquefied natural gas (LNG), natural gas, or electricity were classified as non-solid-fuel users. The survey also asked whether respondents had cooked during the week preceding the interview, and a subset reported their average weekly cooking time. Accordingly, we included an indicator for food preparation during the prior week as a proxy for household air pollution exposure and, in robustness checks, additionally controlled for reported weekly cooking time.

### 2.3 Control variables

The models additionally included socioeconomic and demographic covariates. Although current students were excluded, the education level was retained to capture adult or continuing education. Education was classified into ordered categories: no formal education; primary school; lower middle school; upper middle school; technical/vocational; college or university; and master’s degree or higher. A binary indicator captured current employment status (1 = employed; 0 = unemployed). We also included per capita household income, inflation-adjusted to 2015 values. Other controls were age, marital status, current smoking, pregnancy status, drinking, and urban residence. Given prior evidence linking body mass index (BMI) to blood pressure (23, 24), BMI was included as a covariate in the main analysis.

Table 1 reports summary statistics. Column 1 describes the full sample. Columns 2–4 present outcomes by primary fuel type, and Wald tests assess differences between clean- and solid-fuel users. In unadjusted comparisons, solid-fuel users exhibit lower mean SBP and DBP and are less likely to have hypertension, SBP ≥ 110 mmHg, and SBP ≥ 140 mmHg. Women whose primary fuel is clean are older and of higher socioeconomic status than those who primarily use solid fuels; specifically, they have higher incomes and education levels, are more likely to be employed, and are more likely to reside in urban areas.

**Table 1:** Summary statistics for women using solid fuel as primary fuel and clean fuel as primary fuel.

	Total		Primary fuel: clean		Primary fuel: solid		*t*-tests
	Mean	SD	Mean	SD	Mean	SD	
Outcome variables							
Diastolic Blood Pressure (DBP)	77.89	11.25	78.30	10.97	77.30	11.62	0.995***
Systolic Blood Pressure (SBP)	121.8	19.63	122.50	19.34	120.85	20.00	1.653***
Hypertension	0.219	0.413	0.224	0.417	0.210	0.408	0.014***
SBP above 110 mm Hg	0.630	0.483	0.661	0.473	0.586	0.493	0.075***
SBP above 140 mm Hg	0.231	0.422	0.237	0.425	0.224	0.417	0.013***
Control variables							
Solid Fuel as Primary Fuel	0.414	0.493	0	0	1	0	−1.000
Cooking at Home (Last week)	0.849	0.358	0.827	0.378	0.880	0.325	−0.053***
Time Spent on Cooking (Last week)	65.56	55.36	60.833	53.594	72.249	57.117	−11.416***
Age	49.41	15.34	49.912	15.071	48.698	15.681	1.214***
Married	0.831	0.374	0.836	0.370	0.825	0.380	0.011**
Current Employed	0.541	0.498	0.477	0.499	0.631	0.483	−0.154***
Employed position							
None Position	0		0		0		0
Professional and Technical staff	1.490		1.497		1.449		0.048
Administrator	3.161		3.333		3.077		0.256***
Farmer or Fisherman	5		5		5		0
Worker	6.600		6.550		6.218		0.158***
Gross Household Income	8.777	1.163	9.122	1.103	8.287	1.065	0.836***
Education							
None Education	0		0		0		0
Less Than High School	2.342		1.623		1.450		0.172***
High School or Vocational School	3.629		3.579		3.243		0.034*
College or More	5.516		5.390		5.900		−0.501***
Urban Residence	0.331	0.471	0.445	0.497	0.169	0.375	0.276***
Current smoke	0.038	0.190	0.033	0.178	0.045	0.207	−0.012***
Drink	0.088	0.295	0.940	0.312	0.079	0.270	0.015***
Body Mass Index (BMI)	23.24	4.089	23.584	4.099	22.757	4.025	0.827***
Current Pregnant status	0.010	0.097	0.009	0.094	0.010	0.102	−0.002
Observations	30,844		18,084		12,760		

To further explore potential endogeneity issues, this study presents descriptive statistics for different fuel transition groups. As shown in Table A2, among the groups that did not change fuel, transitioned from solid fuel to clean fuel, and transitioned from clean fuel to solid fuel, income changes were modest, and blood pressure changes were limited, while urban residency showed significant variation. This suggests that migration may be a factor influencing fuel transitions. To control for these potential effects, we included income and urban residency status as control variables in all regression models. Given the small changes in income and blood pressure, we further provide descriptive statistics for the periods before and after the transition. As shown in Table A3, both fuel transitions are associated with a slight increase in blood pressure, which is likely related to age. Since the data collection spans several years, participants’ ages increase over time, and we have accounted for this by including age as a control variable in the models. The group transitioning from solid fuel to clean fuel shows higher urban residency rates and income growth, while the group transitioning from clean fuel to solid fuel exhibits the opposite trend. Consequently, we have controlled for other potentially confounding variables in the models.

**Figure 1:**
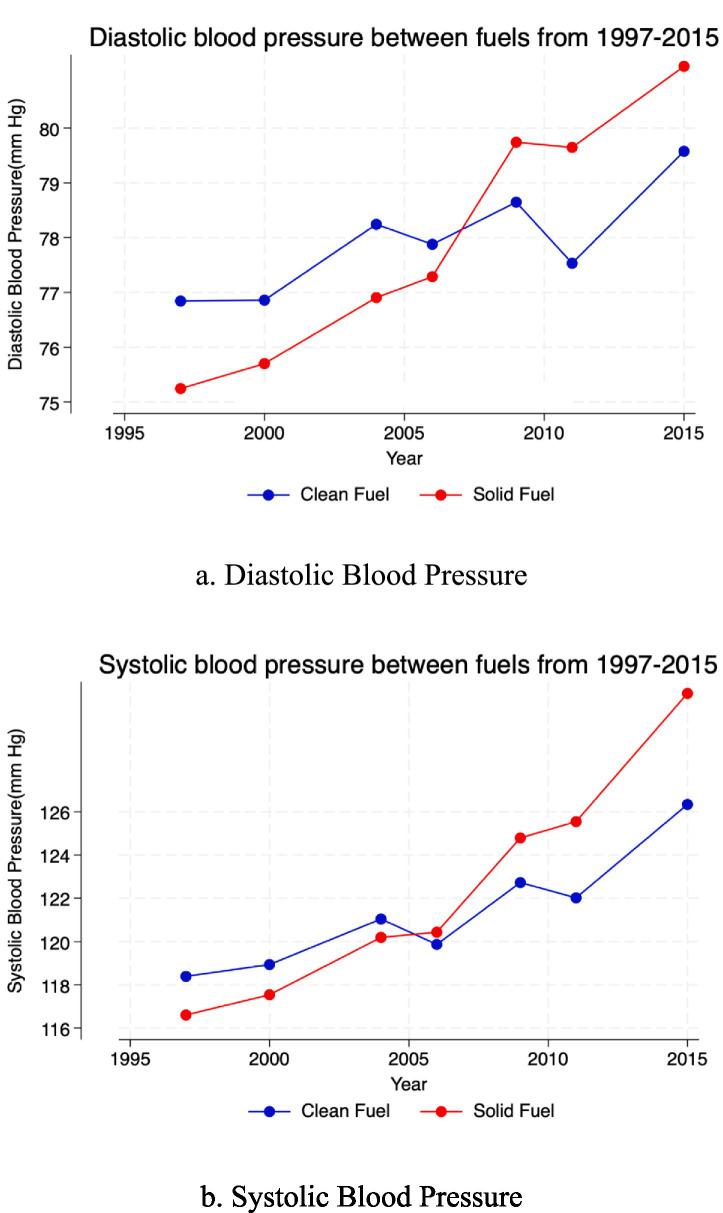
Trends of blood pressures by primary fuel type. **(a)** Diastolic blood pressure. **(b)** Systolic blood pressure.

Figure 1 shows trends in blood pressure over the study period by primary cooking-fuel type. Panels 1a and 1b indicate that both diastolic (DBP) and systolic (SBP) pressures increase steadily over time. Before 2006, women using clean fuels exhibited higher mean DBP; after 2006, DBP rose sharply among solid-fuel users, narrowing the difference. A comparable pattern is observed for SBP. On average, DBP increases by approximately 3–4 mmHg between 1997 and 2015, and SBP by about 7–8 mmHg over the same period.

### 2.4 Empirical framework

The regression model in this paper is as follows:


$$Y_{\mathrm{ict}}=\beta_{1}\text{Solid}\_\text{primar}y_{\mathrm{ict}}+\pi X_{\mathrm{ict}}+\theta_{i}+\delta_{t}+\mu_{\mathrm{ict}}$$

$Y_{\mathrm{ict}}$ represents the indicators for blood pressure of individual i living at c community in wave t . There are five outcome indicators: DBP, SBP, hypertension, SBP above 110 mmHg, and SBP at 140 mmHg. $\text{Solid}\_\text{primar}y_{\mathrm{ict}}$ represents the primary type of fuel used in the household of female i . $X_{\mathrm{ict}}$ are a series of control variables, including cooking behavior, level of education, age, whether employed, married status, smoking and drinking habits, and so on. $\theta_{i}$ and $\delta_{t}$ are individual-, time-, and community-fixed effects. Individual fixed effects are included to account for time-invariant individual unobserved characteristics. Therefore, our identifications do not come from comparing different individuals but rather from the same individuals switching between solid and clean fuels over time. Time-fixed effects capture nationwide shocks that affect both health outcomes and household solid fuel use, such as the national economic crisis and seasonal fluctuations in blood pressure. The month of the survey had a significant impact on the intensity of solid fuel use, as respondents also used it for heating in winter. $\mu_{\mathrm{ict}}$ is an idiosyncratic error term.

**Figure 2:**
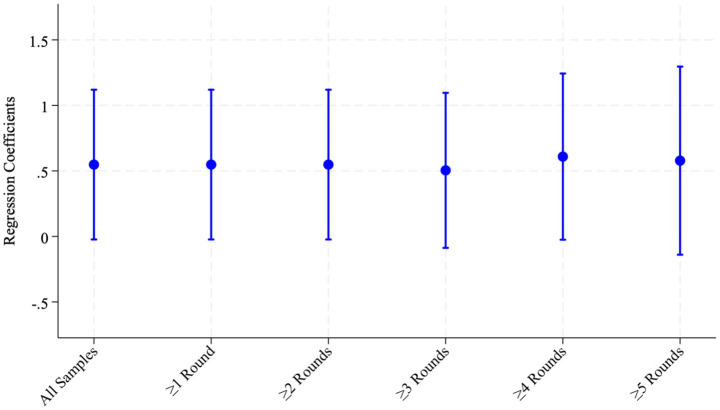
Regression coefficients by different participation rounds.

Our previous discussion noted that our study was limited to assessing the effects of solid fuel use and cooking behavior in a population that changed fuel types during the study period. We predicted that the coefficient $\beta_{1}$ should be positive, implying that the use of solid fuels at home may increase blood pressure. Similarly, because women who cook at home are more likely to be exposed to air pollutants from solid-fuel combustion, the coefficient associated with cooking behavior is also expected to be positive (Figure 2).

## 3 Results

Tables 2–5 present the results. Table 2 reports the main estimates using five outcomes: diastolic blood pressure (DBP), systolic blood pressure (SBP), a binary indicator for hypertension, and indicators for SBP ≥ 110 mmHg and SBP ≥ 140 mmHg. Columns 1–5 show pooled OLS estimates without individual fixed effects, whereas Columns 6–10 present individual fixed-effects (FE) estimates that control for time-invariant, individual-specific heterogeneity.

**Table 2:** Regression results.

Variables	(1)	(2)	(3)	(4)	(5)	(6)	(7)	(8)	(9)	(10)
	DBP	SBP	Hypertension	SBP > 110	SBP > 140	DBP	SBP	Hypertension	SBP > 110	SBP > 140
Primary Fuel: Solid	0.244*	0.930***	0.012**	0.013**	0.006	−0.127	0.548*	0.011	0.001	0.006
	(1.65)	(3.96)	(2.24)	(2.13)	(1.02)	(−0.68)	(1.88)	(1.45)	(0.11)	(0.77)
Control variables	X	X	X	X	X	X	X	X	X	X
Time fixed effects	X	X	X	X	X	X	X	X	X	X
Individual fixed effects						X	X	X	X	X
Province fixed effects	X	X	X	X	X	-	-	-	-	-
Observations	30,844	30,844	30,844	30,844	30,844	26,050	26,050	26,050	26,050	26,050
R-squared	0.182	0.312	0.180	0.227	0.201	0.654	0.719	0.584	0.585	0.617

Robust standard errors in parentheses. ***p < 0.01, **p < 0.05, *p < 0.1. For columns 1 to 5, the results are estimated through pooled OLS models after controlling for age, marital status, cooking status, education level, urban residence, employment status, gross income per capita, smoking, drinking, pregnant status, BMI, time fixed effects, and province fixed effects. For columns 6–10, the coefficient standard errors are robust to heteroscedasticity. For columns 6 to 10, the results are estimated using fixed effects models after controlling for age, marital status, cooking status, education level, urban residence, employment status, gross income per capita, smoking, drinking, pregnant status, BMI, individual fixed effects, and time fixed effects. For columns 6–10, coefficient standard errors are robustly adjusted for potential heteroskedasticity.

**Table 3:** Regression results for rural and urban residence.

Variables	(1)	(2)	(3)	(4)	(5)
	DBP	SBP	Hypertension	SBP > 110	SBP > 140
Panel A: Rural					
Primary Fuel: Solid	−0.224	0.474	0.006	−0.002	0.005
	(−1.05)	(1.43)	(0.70)	(−0.17)	(0.62)
Observations	17,482	17,482	17,482	17,482	17,482
R-squared	0.651	0.711	0.573	0.575	0.602
Panel B: Urban					
Primary Fuel: Solid	0.268	1.051*	0.033**	0.007	0.011
	(0.65)	(1.65)	(2.09)	(0.40)	(0.74)
Observations	7,203	7,203	7,203	7,203	7,203
R-squared	0.683	0.753	0.626	0.624	0.676
Control variables	X	X	X	X	X
Time fixed effects	X	X	X	X	X
Individual fixed effects	X	X	X	X	X

Robust standard errors in parentheses. ***p < 0.01, **p < 0.05, *p < 0.1. Notes: the results are from a fixed effects model after controlling for age, marital status, cooking status, education level, urban residence, employment status, gross income per capita, smoking, drinking, pregnant status, BMI, individual fixed effects, and time fixed effects. All coefficient standard errors are robustly adjusted for potential heteroskedasticity.

**Table 4:** Regression results for urban and rural areas: controlling for housing area.

Variables	(1)	(2)	(3)	(4)	(5)
	DBP	SBP	Hypertension	SBP > 110	SBP > 140
Panel A: Rural					
Primary Fuel: Solid	−0.177	−0.022	0.005	−0.016	0.002
	(−0.56)	(−0.04)	(0.37)	(−1.14)	(0.17)
House Area	−0.001	−0.000	0.000	0.000	−0.000
	(−0.50)	(−0.13)	(0.01)	(0.81)	(−0.22)
Observations	8,890	8,890	8,890	8,890	8,890
R-squared	0.736	0.786	0.676	0.671	0.698
Panel B: Urban					
Primary Fuel: Solid	1.806**	2.344**	0.072**	0.064**	0.041
	(2.45)	(2.15)	(2.52)	(2.04)	(1.53)
House Area	−0.002	0.001	−0.000	−0.000	0.000
	(−0.86)	(0.20)	(−0.14)	(−0.10)	(0.09)
Observations	3,643	3,643	3,643	3,643	3,643
R-squared	0.745	0.808	0.699	0.712	0.747
Control Variables	X	X	X	X	X
Time Fixed Effects	X	X	X	X	X
Individual Fixed Effects	X	X	X	X	X

Robust standard errors in parentheses. ***p < 0.01, **p < 0.05, *p < 0.1. The results are from a fixed effects model after controlling for age, marital status, cooking status, education level, urban residence, employment status, gross income per capita, smoking, drinking, pregnant status, BMI, individual fixed effects, and time fixed effects. All coefficient standard errors are robustly adjusted for potential heteroskedasticity.

**Table 5:** Regression results for working.

Variables	(1)	(2)	(3)	(4)	(5)
	DBP	SBP	Hypertension	SBP > 110	SBP > 140
Panel A: Not working					
Primary Fuel: Solid	−0.134	0.395	0.006	0.005	−0.001
	(−0.36)	(0.65)	(0.37)	(0.33)	(−0.09)
Observations	10,053	10,053	10,053	10,053	10,053
R-squared	0.703	0.752	0.656	0.650	0.691
Panel B: Working					
Primary Fuel: Solid	0.276	0.994***	0.021**	0.015	0.020**
	(1.07)	(2.59)	(2.18)	(1.12)	(2.02)
Observations	13,148	13,148	13,148	13,148	13,148
R-squared	0.702	0.748	0.614	0.624	0.628
Control variables	X	X	X	X	X
Time fixed effects	X	X	X	X	X
Individual fixed effects	X	X	X	X	X

Robust standard errors in parentheses. ***p < 0.01, **p < 0.05, *p < 0.1. The results are from a fixed effects model after controlling for age, marital status, cooking status, education level, urban residence, employment status, gross income per capita, smoking, drinking, pregnant status, BMI, individual fixed effects, and time fixed effects. All coefficient standard errors are robustly adjusted for potential heteroskedasticity.

In Table 2, columns 1 and 6 indicate no statistically significant association between solid-fuel use and diastolic blood pressure (DBP) in pooled OLS or individual fixed-effects (FE) models. Column 2 shows a positive OLS association with systolic blood pressure (SBP); when individual fixed effects are included (column 7), the association remains statistically significant, with an estimated increase of 0.548 mmHg. No significant association with hypertension is detected in either specification (columns 3 and 8). Likewise, FE estimates for SBP ≥ 110 mmHg and SBP ≥ 140 mmHg (columns 9–10) are not statistically significant. Across outcomes, pooled OLS coefficients tend to exceed FE estimates, consistent with an upward bias when individual-specific, time-invariant confounding is not controlled for.

Table 3 re-estimates individual fixed-effects (FE) models separately for rural and urban women and shows stronger associations in urban settings. Panel A indicates no statistically significant association between solid-fuel use and either systolic blood pressure (SBP) or hypertension among rural women. In contrast, Panel B shows that urban women are more sensitive to solid-fuel use: reliance on solid fuels is associated with a 1.051 mmHg higher SBP (*p* < 0.10) and a 3.3% higher probability of hypertension (*p* < 0.05). A plausible explanation is household layout: rural kitchens are often separate from living areas, whereas urban kitchens are typically integrated into them, increasing exposure to combustion pollutants and, consequently, health risks.

To further investigate the reasons behind the urban–rural differences, we controlled for housing area and re-ran the regression analysis. The results, shown in Table 4, reveal that the effect of solid fuel use is stronger in the urban sample, while it remains insignificant in the rural sample. This finding further supports the manuscript’s argument that urban housing tends to be more compact and that an integrated kitchen design may exacerbate the health impacts of solid fuel use. In contrast, rural housing is generally more spacious, with kitchens separated from living areas, reducing the health risks associated with solid fuel use.

Table 5 re-estimates individual fixed-effects (FE) models after stratifying the sample by employment status. Results indicate stronger associations among currently employed women. Panel A shows no statistically significant association between primary solid-fuel use and any outcome among nonworking women. In contrast, Panel B shows that, among employed women, solid-fuel use is associated with a 0.994 mmHg higher systolic blood pressure (SBP; *p* < 0.01), a 2.2% higher probability of hypertension (*p* < 0.05), and a 2.1% higher probability of SBP ≥ 140 mmHg (*p* < 0.05). A plausible explanation is that these employed women face not only work-related stress but also the responsibility of cooking at home, resulting in dual pressures (37, 38). In this context, systolic blood pressure tends to increase (39) and is associated with hypertension and cardiovascular diseases (40).

Table 6 re-estimates individual fixed-effects (FE) models by age group: women <60 years (Group A) and women≥60 years (Group B). Results indicate stronger associations among women aged ≥60. In Group A, solid-fuel use is not significantly associated with any outcome other than systolic blood pressure (SBP), for which a statistically significant association is observed. In Group B, solid-fuel use is associated with a 4.6% higher probability of hypertension (*p* < 0.05) and a 3.4% higher probability of SBP ≥ 140 mmHg (*p* < 0.10). We offer two potential explanations for these results. First, prior to 2015, the majority of women in China reached retirement age, resulting in more time spent at home than working women, which increased their exposure to solid fuel combustion (41). Second, individuals aged 60 of age and above are more sensitive to environmental exposures, making them more susceptible to negative health impacts (42).

**Table 6:** Regression results for women below 60 and above 60 years of age.

Variables	(1)	(2)	(3)	(4)	(5)
	DBP	SBP	Hypertension	SBP > 110	SBP > 140
Panel A: Age under 60 years					
Primary Fuel: Solid	0.176	0.777**	0.007	0.004	0.003
	(0.81)	(2.43)	(0.91)	(0.36)	(0.35)
Observations	18,792	18,792	18,792	18,792	18,792
R-squared	0.679	0.714	0.583	0.596	0.607
Panel B: Age 60 and above					
Primary Fuel: Solid	0.172	0.063	0.046**	0.020	0.034*
	(0.37)	(0.08)	(2.16)	(1.13)	(1.69)
Observations	6,105	6,105	6,105	6,105	6,105
R-squared	0.735	0.750	0.662	0.636	0.692
Control variables	X	X	X	X	X
Time fixed effects	X	X	X	X	X
Individual fixed effects	X	X	X	X	X

Robust standard errors in parentheses. ***p < 0.01, **p < 0.05, *p < 0.1. The results are from a fixed effects model after controlling for age, marital status, cooking status, education level, urban residence, employment status, gross income per capita, smoking, drinking, pregnancy status, BMI, individual fixed effects, and time fixed effects. All coefficient standard errors are robustly adjusted for potential heteroskedasticity.

Table 7 reports robustness checks. Part A redefines hypertension using the American Heart Association (AHA) criteria—diastolic blood pressure (DBP) ≥ 80 mmHg or systolic blood pressure (SBP) ≥ 130 mmHg—and re-estimates the models with this binary outcome. Panel B divides solid fuels into biomass and coal, with results showing a stronger association between coal and both systolic blood pressure and hypertension. Panel C applies a 1% winsorization to both the core explanatory and outcome variables. Panel D replaces the binary cooking variable with cooking time and includes the square of cooking time to test for non-linear relationships. The final estimates from all four panels are broadly consistent with those in Table 2. Additionally, we included the lagged effect of the dependent variable for the next period, used self-reported health status as a placebo test for the dependent variable, conducted subgroup regressions by individual participation rounds, adjusted standard errors for individual clustering, and incorporated tests for household fixed effects. All results remain consistent with the Appendix.

**Table 7:** Robustness tests.

Variables	(1)	(2)	(3)	(4)	(5)
	DBP	SBP	Hypertension	SBP > 110	SBP > 140
Panel A: Robustness Check 1					
Primary Fuel: Solid	−0.127	0.548*	−0.006	0.001	0.006
	(−0.68)	−1.88	(−0.71)	−0.11	−0.77
Observations	26,050	26,050	26,050	26,050	26,050
Panel B: Robustness Check 2					
Coal	−0.12	0.632*	0.016*	0.001	0.009
	(−0.53)	−1.77	−1.75	−0.13	−1.02
Biomass	−0.194	0.3	0.002	−0.001	−0.001
	(−0.81)	−0.79	−0.25	(−0.06)	(−0.16)
Observations	25,962	25,962	25,962	25,962	25,962
Panel C: Robustness Check 3					
Primary Fuel: Solid	−0.125	0.562**	0.011	0.001	0.006
	(−0.71)	−2	−1.45	−0.11	−0.77
Observations	26,050	26,050	26,050	26,050	26,050
Panel D: Robustness Check 4					
Primary Fuel: Solid	−0.124	0.548*	0.011	0.001	0.006
	(−0.67)	−1.88	−1.46	−0.12	−0.78
Cooking time	−0.004	0.002	0	0	0
	(−1.61)	−0.53	(−1.19)	(−0.78)	(−0.86)
Cooking time^2^	0.000*	0	0	0	0
	−1.67	−0.05	−1.45	−0.81	−1.29
Observations	26,050	26,050	26,050	26,050	26,050
Control Variables	X	X	X	X	X
Time Fixed Effects	X	X	X	X	X
Individual Fixed Effects	X	X	X	X	X

Robust standard errors in parentheses. ***p < 0.01, **p < 0.05, *p < 0.1. For all models, control variables include age, marital status, cooking status, education level, urban residence, employment status, gross income per capita, smoking status, drinking status, pregnancy status, BMI, individual fixed effects, and time fixed effects. Standard errors are clustered at the individual level. All coefficient standard errors are robustly adjusted for potential heteroskedasticity. For panel a robustness check 1, we used the average of DBP and SBP across three blood pressure measurements instead of the average of the second and third measurements. We also use the American Heart Association’s definition of hypertension. For robustness check 2 in Panel B, we decompose the fuel types into biomass and coal. For robustness check 3 in Panel C, we apply 1% winsorization to both the core explanatory and outcome variables, then re-estimate. For robustness check 4 in Panel D, we replace the binary cooking indicator with cooking time as a control variable and include the squared term of cooking time to test for non-linear relationships.

## 4 Discussion

This study provides new evidence on the association between household solid-fuel use and elevated blood pressure. Our results indicate that pooled OLS estimates tend to overstate the association relative to individual fixed-effects (FE) estimates, consistent with an upward bias from unobserved, time-invariant confounding. In contrast, estimates for diastolic blood pressure (DBP) are not statistically significant, which may reflect the lower temporal sensitivity of DBP relative to systolic blood pressure (SBP) (22, 30, 32, 43). Accordingly, our findings differ from several prior studies in China that reported stronger associations between air pollution and diastolic blood pressure (DBP) (23, 29).

On average, the association between primary solid-fuel cooking and systolic blood pressure (SBP) is modest—approximately 0.548 mmHg. First, even small increases in blood pressure are associated with higher mortality and a greater incidence of cardiovascular diseases (44–46). For example, a 1 mmHg increase in SBP is associated with approximately a 2% increase in all-cause mortality (44, 47). A study by Ettehad et al. (48) found that a 10 mmHg reduction in systolic blood pressure (SBP) corresponds to a 20% decrease in cardiovascular risk. Based on this, we estimate that a 0.548 mmHg increase in SBP could lead to a 1.1% rise in cardiovascular risk. Second, this study focuses only on the use of solid fuels for cooking, resulting in a more conservative effect estimate, mainly because of the shorter exposure time associated with cooking. If solid fuels are used for heating, the exposure time is longer, potentially leading to more significant effects on blood pressure. However, even with cooking exposure, we still observe significant effects on blood pressure. Third, given the large populations in China and globally that continue to rely on solid fuels, even a modest 0.548 mmHg increase in SBP could have a considerable public health impact. Finally, compared with other risk factors, high dietary salt intake significantly raises both SBP and diastolic blood pressure (DBP) in salt-sensitive individuals, but not in salt-insensitive individuals (49). Regular aerobic exercise, such as 30 min per week, can reduce SBP by 1.78 mmHg (50). However, a study found that increased sedentary time did not significantly raise SBP in older adults (51). These significant effects may be observed only in specific groups. Similarly, the impact of cooking with solid fuels also warrants attention. In conclusion, the health impacts of cooking with solid fuels should not be overlooked in public health discussions.

This study has several limitations. First, due to a lack of relevant data, it does not include a cost-effectiveness analysis of fuel transition. The main contribution of this research is the identification of potential cardiovascular health risks associated with solid fuel use in women and a suggestion that future studies incorporate economic evaluations at the intervention level. Second, because China’s large-scale coal-to-gas policy began in 2017 and the current CHNS data only extends to 2015, the research sample in this study is minimally affected by this policy. However, future research will attempt to incorporate the coal-to-gas policy as an instrumental variable in the analysis. Third, identifying variation is limited: many households do not change their primary cooking fuel over time, so fixed-effects estimates are identified primarily from switchers. Consequently, the estimates pertain to women who changed their primary cooking fuel during the study period and may not generalize to non-switchers.

Despite study limitations, this analysis provides credible evidence of the association between household solid-fuel use and blood pressure among Chinese women, leveraging individual fixed-effects models. To advance understanding of indoor air pollution’s health impacts, future research should examine differential associations between systolic and diastolic blood pressure and clarify the biological and behavioral pathways through which combustion-related pollutants may affect cardiovascular and other health outcomes. From a policy perspective, the findings support targeted actions by governments and public health agencies to promote the adoption of clean cooking fuels and technologies; such measures are likely to yield meaningful health benefits for women.

## Data Availability

Publicly available datasets were analyzed in this study. The data used in this study is from the China Health and Nutrition Survey (CHNS), which is managed by the Carolina Population Center at the University of North Carolina at Chapel Hill. The dataset is publicly available and can be accessed through the following link: CHNS Data Download Page. Repository Name: Carolina Population Center at the University of North Carolina at Chapel Hill Accession Number: The CHNS dataset includes multiple waves of data collection. The specific waves used in this study are from 1997 to 2015. Each wave has its own dataset identifier, which can be found on the CHNS website.
