# Supplementary material for: Burning health inequity: blood pressure effects of household cooking with solid fuels among Chinese women
Source: Front Public Health. 2025 Dec 12;13:1683629. doi: 10.3389/fpubh.2025.1683629 (PMC12740932; doi:10.3389/fpubh.2025.1683629)
Supplement: Supplementary file 1 [file Table_1.docx]

**Supplementary file**

Table A1 Selected Study of the impacts of household air pollution on blood pressures

Part 1: The Impact of Specific Pollutants on Blood Pressure

| Study | Location;Yeas | Sample size | Pollutant measured? | Effects |
| --- | --- | --- | --- | --- |
| Clark et al.(2011) | Granada, Nicaragua (Summer 2008) | 124 females | Yes. fine particulate matter and CO. | No significant increase of SBP with rise of CO concentrations among overall population. However, among obese people, higher SBP are observed when CO concentration was high |
| Quinn et al., (2016) | Ghana (2014) | 817 adult pregnant females | Yes. CO | HAP pollution is associated with higher DBP among pregnant female in their middle pregnant stages. |
| Norris et al (2016) | India (winter 2011; summer 2012) | 45 cooking females | Yes. Black Carbon | Cooking with solid fuel is associated with acute increase of SBP. No evidence between cooking with solid fuel and changes of DBP. |
| Baumgartner et al., (2011) | Yunnan, China (winter 2008; summer 2009) | 280 women | Yes. PM2.5 | Increase of PM2.5 level is associated with significant increase of SBP and small increase of DBP. |
| Baumgartner et al., (2018) | SiChuan, China (Summer 2014; Winter 2014) | 205 rural women | Yes PM2.5 and Black Carbon | Increase of air pollutants such as PM2.5 and Black Carbon are associated with rise of brachial and aortic blood pressures both for SBP and DBP. Higher level air pollutant is also associated with higher central hemodynamics, which result in higher risk of CVD. |
| Olsen et al., (2014) | Western Copenhagen Area, Denmark (2013) | 81 nonsmoking people | Yes. Particle concentration and ultrafine particles | Indoor PNC are positively association with higher DBP and SBP increase, a one 10^3^/cm^3^ increase of PNC is associated with 2.4-2.6 mm Hg increase for both DBP and SBP. |
| Baumgartner et al. (2014) | Yunnan, China (2008-2009) | 280 Chinese rural women | Yes. Black carbons, PM_2.5_ mass, and water-soluble organic mass | Black carbon is associated with 4.3 mm Hg higher SBP and the effects of black carbon on SBP is two or three times larger than that of PM_2.5_ mass, and water-soluble organic mass. |
| Ye et al.(2022) | India(HAPIN) | 799 pregnant women | Yes.PM2.5, BC, and CO | Use of solid fuels by pregnant women may raise blood pressure。 |

Part 2: the Impact of Cooking Fuel/Stoves on Blood Pressure

| Study | Location;Yeas | Sample size | Pollutant measured? | Effects |
| --- | --- | --- | --- | --- |
| Nie (2016) | Nine Province^[[1]](#footnote-0)^, China (1991-2009) | 15,539 rural women | No. | liquefied petroleum gas (LPG) users have lower blood pressures comparing to wood/straw and coal users. |
| Alexander et al. (2017) | Ibadan, Nigeria (Summer 2013-Fall 2015) | 324 pregnant females | No. | For females who were assigned with clean stoves, their DBP is 3 mm Hg lower than those who still use their old wood or Kerosene stoves in late gestation stages. |
| Clark et al.(2013) | Granada, Nicaragua (Summer 2008 and Summer 2009) | 79 females | Yes. PM2.5 and CO | Improved stove reduces both CO and PM concentration. However, improved stove did not reduce the SBP among the entire population. SBP for females above 40 or obese females were saw a significant decrease after stove improvement. |
| Arku et al., (2018) | Ten countries^[[2]](#footnote-1)^ | 77,605 females | No. | Cooking with polluting fuels as major fuel was associated with very small increase of systolic blood pressure and odds of hypertension. SBP 0.58 mm Hg. |
| Yan et al. (2016) | China (2009) | 4594 health adults | No. | Use of solid fuel was associated with elevated blood pressures and higher chance of having hypertension. |
| Lee et al. (2012) | Shanghai, China (2007-2009) | 14,068 adults | No. | In-home solid fuel use was associated with increased risk for hypertension. Hypertension risk increased with the duration of solid fuel exposure. |
| McCracken et al. (2007) | Guatemalan (March 2003-2005) | 120 females above 38 years old | Yes. PM2.5 | Using of improved stove was associated with lower daily PM2.5 exposure and lower SBP and DBP. 3.7 lower mm Hg for SBP and 3.0 mm Hg for DBP. |
| Alexander et al. (2015) | Bolivia (fall 2009-fall 2010) | 28 females | Yes. PM. | Improved stove user was associated with lower PM level and significant decrease of SBP 0.5 mm Hg, but no significant decrease of DBP. They also reports a significant decrease of SBP for females over 50 |

**Table A2 Descriptive Statistics for Different Fuel Transition Groups**

|  | No Fuel Change | | Solid Fuel to Clean Fuel | | Clean Fuel to Solid Fuel | |
| --- | --- | --- | --- | --- | --- | --- |
|  | Mean | SD | Mean | SD | Mean | SD |
| **Outcome Variables** |  |  |  |  |  |  |
| Diastolic Blood Pressure (DBP) | 77.81218 | 11.21416 | 78.3092 | 11.33201 | 78.48026 | 11.77506 |
| Systolic Blood Pressure (SBP) | 121.7264 | 19.63926 | 122.3447 | 19.68473 | 122.4905 | 19.3418 |
| Hypertension | 0.2178591 | 0.4127989 | 0.2223027 | 0.4158687 | 0.2270311 | 0.419072 |
| SBP above 110 mm Hg | 0.6301513 | 0.4827726 | 0.6292542 | 0.483092 | 0.6188307 | 0.4858585 |
| SBP above 140 mm Hg | 0.2282085 | 0.4196855 | 0.2494569 | 0.432777 | 0.2536067 | 0.4352404 |
|  |  |  |  |  |  |  |
| **Control Variables** |  |  |  |  |  |  |
| Solid Fuel as Primary Fuel | 0.4266766 | 0.4946037 | 0.0083273 | 0.0908897 | 1 | 0 |
| Cooking at Home (Last week) | 0.8408369 | 0.3658349 | 0.8960898 | 0.3051993 | 0.9141989 | 0.2801765 |
| Time Spent on Cooking (Last week) | 64.85911 | 55.54082 | 69.75742 | 55.14025 | 70.90357 | 51.45616 |
| Age | 49.13825 | 15.52807 | 51.24001 | 14.02083 | 51.07935 | 13.64069 |
| Married | 0.8286195 | 0.3768482 | 0.8432295 | 0.3636501 | 0.8625664 | 0.3444353 |
| Current Employed | 0.5377919 | 0.498579 | 0.5666184 | 0.4956318 | 0.5482156 | 0.4978589 |
| Employed Position |  |  |  |  |  |  |
| None Position | 0 |  | 0 |  | 0 |  |
| Professional and Technical staff | 1.49406 |  | 1.43299 |  | 1.444444 |  |
| Administrator | 3.166954 |  | 3.13254 |  | 3.125475 |  |
| Farmer or Fisherman | 5 |  | 5 |  | 5 |  |
| Worker | 6.632246 |  | 6.273356 |  | 6.251908 |  |
| Gross Household Income | 8.793162 | 1.178615 | 8.648794 | 1.05689 | 8.708835 | 1.025921 |
| Education |  |  |  |  |  |  |
| None Education | 0 |  | 0 |  | 0 |  |
| Less Than High School | 1.563089 |  | 1.464907 |  | 1.460648 |  |
| High School or Vocational School | 3.625478 |  | 3.646643 |  | 3.701149 |  |
| College or More | 5.518295 |  | 5.375 |  | 5.5 |  |
| Urban Residence | 0.3460116 | 0.4757058 | 0.2143374 | 0.4104362 | 0.2687927 | 0.4435003 |
| Current smoke | 0.0365029 | 0.1875413 | 0.0416365 | 0.1997933 | 0.0523918 | 0.2229005 |
| Drink | 0.0889221 | 0.298477 | 0.0803765 | 0.2719245 | 0.0789674 | 0.2697902 |
| Body Mass Index (BMI) | 23.23318 | 4.084731 | 23.37036 | 4.026675 | 23.15655 | 4.296108 |
| Current Pregnant | 0.0101999 | 0.10048 | 0.0050688 | 0.0710276 | 0.0045558 | 0.0673684 |
| Observations | 26765 |  | 2762 |  | 1317 |  |

**Table A3 Descriptive Statistics Before and After Different Fuel Transition Patterns**

|  | Solid Fuel to Clean Fuel | | | | | Clean Fuel to Solid Fuel | | | | |
| --- | --- | --- | --- | --- | --- | --- | --- | --- | --- | --- |
|  | t0-2 | t0-1 | t0 | t0+1 | t0+2 | t0-2 | t0-1 | t0 | t0+1 | t0+2 |
|  | Mean | Mean | Mean | Mean | Mean | Mean | Mean | Mean | Mean | Mean |
| Outcome Variables |  |  |  |  |  |  |  |  |  |  |
| Diastolic Blood Pressure (DBP) | 76.706 | 78.339 | 78.165 | 79.453 | 79.876 | 76.799 | 77.689 | 77.543 | 79.033 | 81.509 |
| Systolic Blood Pressure (SBP) | 118.900 | 121.427 | 122.242 | 124.294 | 124.678 | 117.920 | 120.861 | 121.152 | 123.948 | 126.139 |
| Hypertension | 0.159 | 0.223 | 0.223 | 0.234 | 0.278 | 0.195 | 0.197 | 0.221 | 0.239 | 0.287 |
| SBP above 110 mm Hg | 0.575 | 0.591 | 0.629 | 0.660 | 0.688 | 0.552 | 0.588 | 0.563 | 0.656 | 0.731 |
| SBP above 140 mm Hg | 0.177 | 0.245 | 0.250 | 0.275 | 0.302 | 0.195 | 0.218 | 0.250 | 0.263 | 0.306 |
|  |  |  |  |  |  |  |  |  |  |  |
| Control Variables |  |  |  |  |  |  |  |  |  |  |
| Solid Fuel as Primary Fuel | 0.018 | 0.006 | 0.009 | 0.004 | 0.010 | 1.000 | 1.000 | 1.000 | 1.000 | 1.000 |
| Cooking at Home (Last week) | 0.969 | 0.913 | 0.885 | 0.875 | 0.907 | 0.931 | 0.895 | 0.859 | 0.947 | 0.954 |
| Time Spent on Cooking (Last week) | 76.354 | 72.251 | 69.593 | 67.887 | 64.283 | 82.977 | 70.261 | 63.118 | 73.269 | 79.620 |
| Age | 47.477 | 51.349 | 50.974 | 52.456 | 53.057 | 47.061 | 49.380 | 48.806 | 53.220 | 53.344 |
| Married | 0.916 | 0.892 | 0.816 | 0.857 | 0.898 | 0.874 | 0.882 | 0.802 | 0.893 | 0.861 |
| Current Employed | 0.650 | 0.622 | 0.563 | 0.460 | 0.551 | 0.586 | 0.466 | 0.517 | 0.598 | 0.546 |
| Employed Position |  |  |  |  |  |  |  |  |  |  |
| None Position | 0.000 | 0.000 | 0.000 | 0.000 | 0.000 | 0.000 | 0.000 | 0.000 | 0.000 | 0.000 |
| Professional and Technical staff | 1.333 | 1.667 | 1.475 | 1.304 | 1.400 | 1.500 | 1.261 | 1.615 | 1.429 | 2.000 |
| Administrator | 3.097 | 3.087 | 3.132 | 3.250 | 3.163 | 3.054 | 3.264 | 3.219 | 3.095 | 3.058 |
| Farmer or Fisherman | 5.000 |  |  |  | 5.000 |  | 5.000 | 5.000 |  |  |
| Worker | 6.667 | 6.435 | 6.199 | 6.257 | 6.190 | 6.200 | 6.265 | 6.146 | 6.367 | 6.667 |
| Gross Household Income | 8.372 | 8.503 | 8.639 | 8.941 | 8.757 | 8.547 | 8.729 | 8.708 | 8.616 | 8.975 |
| Education |  |  |  |  |  |  |  |  |  |  |
| None Education | 0.000 | 0.000 | 0.000 | 0.000 | 0.000 | 0.000 | 0.000 | 0.000 | 0.000 | 0.000 |
| Less Than High School | 1.467 | 1.399 | 1.486 | 1.506 | 1.431 | 1.367 | 1.533 | 1.514 | 1.411 | 1.462 |
| High School or Vocational School | 3.471 | 3.667 | 3.665 | 3.655 | 3.684 | 3.667 | 3.661 | 3.788 | 3.567 | 3.692 |
| College or More | 6.000 | 6.000 | 5.240 | 5.250 | 6.000 | 6.000 | 5.286 | 5.500 |  |  |
| Urban Residence | 0.119 | 0.146 | 0.206 | 0.457 | 0.180 | 0.299 | 0.479 | 0.359 | 0.139 | 0.148 |
| Current smoke | 0.062 | 0.050 | 0.032 | 0.053 | 0.054 | 0.046 | 0.067 | 0.046 | 0.047 | 0.074 |
| Drink | 0.066 | 0.074 | 0.074 | 0.125 | 0.102 | 0.115 | 0.080 | 0.078 | 0.081 | 0.065 |
| Body Mass Index (BMI) | 23.390 | 23.044 | 23.297 | 23.905 | 23.736 | 22.730 | 23.442 | 22.531 | 23.325 | 23.567 |
| Current Pregnant | 0.004 | 0.000 | 0.006 | 0.008 | 0.010 | 0.011 | 0.004 | 0.006 | 0.002 | 0.009 |
| Observations | 226 | 323 | 1,631 | 265 | 205 | 87 | 238 | 348 | 468 | 108 |

**Table A4 Regression results for lagged effects**

|  | (1) | (2) | (3) | (4) | (5) |
| --- | --- | --- | --- | --- | --- |
| VARIABLES | DBP(t+1) | SBP(t+1) | Hypertension(t+1) | SBP>110(t+1) | SBP>140(t+1) |
|  |  |  |  |  |  |
| solidfuel | -0.118 | 0.111 | 0.008 | -0.004 | 0.001 |
|  | (-0.51) | (0.30) | (0.86) | (-0.39) | (0.12) |
| Constant | 284.169** | 121.966 | 2.169 | 3.512 | 0.813 |
|  | (2.21) | (0.60) | (0.41) | (0.59) | (0.16) |
|  |  |  |  |  |  |
| Observations | 17,815 | 17,815 | 17,815 | 17,815 | 17,815 |
| R-squared | 0.657 | 0.720 | 0.599 | 0.583 | 0.632 |
| Control | YES | YES | YES | YES | YES |
| Time FE | YES | YES | YES | YES | YES |
| Individual FE | YES | YES | YES | YES | YES |

**Table A5 Regression results for lagged effects**

|  | (1) |
| --- | --- |
| VARIABLES | selfhealth |
|  |  |
| solidfuel | 0.029 |
|  | (1.45) |
| Constant | 2.512** |
|  | (2.32) |
|  |  |
| Observations | 16,187 |
| R-squared | 0.575 |
| Control | YES |
| Time FE | YES |
| Individual FE | YES |

**Table A6 Regression Results for Changing Clustering Standard Errors**

|  | (1) | (2) | (3) | (4) | (5) |
| --- | --- | --- | --- | --- | --- |
| VARIABLES | DBP | SBP | Hypertension | SBP>110 | SBP>140 |
|  |  |  |  |  |  |
| solidfuel | -0.127 | 0.548* | 0.011 | 0.001 | 0.006 |
|  | (-0.69) | (1.89) | (1.49) | (0.11) | (0.79) |
| Constant | 75.535*** | 144.060*** | -0.073 | 1.224* | 0.149 |
|  | (4.21) | (6.46) | (-0.13) | (1.81) | (0.26) |
|  |  |  |  |  |  |
| Observations | 26,050 | 26,050 | 26,050 | 26,050 | 26,050 |
| R-squared | 0.654 | 0.719 | 0.584 | 0.585 | 0.617 |
| Control | YES | YES | YES | YES | YES |
| Time FE | YES | YES | YES | YES | YES |
| Individual FE | YES | YES | YES | YES | YES |

**Table A7 Regression Results for Including Household Fixed Effects**

|  | (1) | (2) | (3) | (4) | (5) |
| --- | --- | --- | --- | --- | --- |
| VARIABLES | DBP | SBP | Hypertension | SBP>110 | SBP>140 |
|  |  |  |  |  |  |
| solidfuel | -0.130 | 0.541* | 0.012 | 0.001 | 0.006 |
|  | (-0.69) | (1.83) | (1.56) | (0.07) | (0.84) |
| Constant | 74.560*** | 147.158*** | 0.015 | 1.300* | 0.227 |
|  | (3.98) | (6.44) | (0.02) | (1.87) | (0.38) |
|  |  |  |  |  |  |
| Observations | 25,921 | 25,921 | 25,921 | 25,921 | 25,921 |
| R-squared | 0.657 | 0.720 | 0.586 | 0.589 | 0.619 |
| Control | YES | YES | YES | YES | YES |
| Time FE | YES | YES | YES | YES | YES |
| Individual FE | YES | YES | YES | YES | YES |
| Family | YES | YES | YES | YES | YES |

1. Liaoning, Heilongjiang, Jiangsu, Shandong, He’nan, Hubei, Hu’nan, Guangxi and Guizhou [↑](#footnote-ref-0)
2. Albania, Armenia, Azerbaijan, Bangladesh, Benin, Ghana, Kyrgyzstan, Lesotho, Namibia, and Peru [↑](#footnote-ref-1)
